# Anodal Transcranial Direct Current Stimulation over the Vertex Enhances Leg Motor Cortex Excitability Bilaterally

**DOI:** 10.3390/brainsci9050098

**Published:** 2019-04-29

**Authors:** Soumya Ghosh, David Hathorn, Jennifer Eisenhauer, Jesse Dixon, Ian D. Cooper

**Affiliations:** 1 Centre for Neuromuscular and Neurological Disorders, Perron Institute for Neurological and Translational Science, University of Western Australia, QEII Medical Centre, Nedlands, WA 6009, Australia; dave.hathorn@mswa.org.au (D.H.); jenny.eisenhauer@perron.uwa.edu.au (J.E.); Jesse.Dixon@health.wa.gov.au (J.D.); 2 Department of Physiotherapy, Sir Charles Gairdner Hospital, Hospital Avenue, Nedlands, WA 6009, Australia; Ian.Cooper@health.wa.gov.au

**Keywords:** transcranial direct current stimulation, leg motor cortex, corticospinal excitability

## Abstract

In many studies, anodal transcranial Direct Current Stimulation (tDCS) is applied near the vertex to simultaneously facilitate leg motor cortex (M1) of both hemispheres and enhance recovery of gait and balance in neurological disorders. However, its effect on the excitability of leg M1 in either hemisphere is not well known. In this double-blind sham-controlled study, corticospinal excitability changes induced in leg M1 of both hemispheres by anodal (2 mA for 20 minutes) or sham tDCS (for 20 min) over the vertex were evaluated. Peak amplitudes of Transcranial Magnetic Stimulation (TMS) induced motor evoked potentials (MEPs) were measured over the contralateral Tibialis Anterior (TA) muscle before and up to 40 min after tDCS in 11 normal participants. Analysis of data from all participants found significant overall increase in the excitability of leg M1 after tDCS. However, in individual subjects there was variability in observed effects. In 4 participants, 20 min of tDCS increased mean MEPs of TAs on both sides; in 4 participants there was increased mean MEP only on one side and in 3 subjects there was no change. It’s not known if the benefits of tDCS in improving gait and balance are dependent on excitability changes induced in one or both leg M1; such information may be useful to predict treatment outcomes.

## 1. Introduction

Balance and mobility are impaired in many neurological disorders, including stroke, multiple sclerosis and Parkinson’s disease. Non-invasive brain stimulation using Transcranial Magnetic Stimulation (TMS) or transcranial Direct Current Stimulation (tDCS), with or without physical therapy, is a promising tool for improving gait and balance [1,2,3,4,5,6,7]. However, results of such treatments are variable and it’s important to find markers that may predict response [7,8].

Transcranial direct current stimulation (tDCS) is a relatively inexpensive device which is increasingly being used to study brain function in normal humans and explore beneficial effects in neurological and psychiatric disorders [9,10,11]. Furthermore, tDCS has been shown to induce prolonged changes in cortical excitability in the motor cortex and other cortical areas [9,12]. However, post stimulation effects of tDCS and other non-invasive brain stimulations (e.g., paired associative stimulation, theta burst stimulation) have been shown to be quite variable between individuals [13,14,15]. Recent studies have explored factors that could influence efficacy of tDCS in order to help design better protocols [16,17,18].

The after effect of tDCS is dependent on the polarity, duration and density of the applied current, and earlier studies evaluated effects on the hand M1 [9,12]. The effects of tDCS (after its termination) can be monitored by TMS evoked Motor Evoked Potentials (MEPs) and depend on changes in the cortex as well as in spinal circuits [19,20,21]. Anodal tDCS applied over the hand M1 increases cortical excitability while cathodal tDCS reduces cortical excitability [9,11].

Later studies have shown different effects when tDCS is applied to leg M1 [15,22,23]. Cathodal tDCS applied to the hand M1 results in reduced or increased excitability depending on the intensity of the applied current [24], while cathodal stimulation applied over the leg M1 has minimal or no effect [22,25]. Anodal tDCS increases excitability of the underlying M1 when applied to hand or leg M1, but some studies suggest that the stimulus intensity required may be higher for the leg M1 [9,12,22,23]. In addition, due to the proximity of the leg M1s of the 2 hemispheres, anodal stimulation applied over one hemisphere reduces excitability of the opposite leg M1 [23]. Differences in tDCS effects on leg vs. hand M1 may be related to differences in the orientation and position of leg vs. hand representations in M1, resulting in differential effects in cortical laminae [26,27,28].

The effects of tDCS of the leg M1 have been studied by applying the stimulus to the leg M1 of one hemisphere at the optimum position from which TMS evokes maximal MEPs in the contralateral leg muscle [1,2,15,21]. However, many studies assessing the therapeutic benefit of anodal tDCS in improving gait or balance have applied the stimulus over the midline close to the vertex (likely between the optimal positions in each hemisphere) to simultaneously facilitate leg M1 of both hemispheres [4,5,6]. In these studies, the cathode was applied to the right supraorbital region [6] or inion [3,4,5]. The effects of tDCS applied to the midline have not been well studied [4] and it’s not known if there is excitation or inhibition of the leg M1 of either hemisphere or variable effects between individuals. Therefore, as part of our study of effects of tDCS in gait and balance we explored the effects of anodal tDCS applied to the midline near the vertex of normal participants.

## 2. Methods

### 2.1. Participants

Eleven healthy individuals (5 female, 6 male), aged 23–66 (mean age 38 years), with no known neurological conditions, participated in the study. All subjects denied the use of any regular medication, drinking alcohol in the previous 24 h, and reported to have had ≥5 h of sleep the previous night. Subjects were tested with anodal tDCS and sham stimulation for 20 min in a pseudo randomized manner. The minimum period between sessions for any one subject was 1 week. All experiments, except one (which was done in the morning), were done in the late morning and early afternoon period.

All participants provided signed informed consent and the study protocol was approved by the Human Research Ethics Committee at Sir Charles Gairdner Group in Western Australia (HREC 2014-026). Prior to assessment, participants were screened for any contraindications to TMS and tDCS, such as the presence of implantable devices or cardiac pacemakers and any history of seizures or epilepsy [29]. All subjects were blinded to the treatment condition. All subjects were right handed.

### 2.2. Experimental Protocol

Subjects were seated in a comfortable chair with their legs extended and resting on another chair for support. Electromyography was recorded from tibialis anterior (TA) muscles of both legs. The vertex was taken as the intersection between the interaural and nasion-inion lines. A tight-fitting EEG cap (EASY CAP; EASYCAP GmbH, Wörthsee, Germany) was fitted and used to localize the hotspot over the lower limb representation of primary motor area (M1) for each TA muscle and to ensure quick and accurate reference. The cap was taken off for tDCS application and reapplied for TMS studies. The position of the cap in relation to the vertex and hotspots were rechecked before starting post tDCS TMS testing and confirmed between stimulation trials throughout. Investigators performing and analyzing the TMS evaluation were blinded to the treatment condition.

### 2.3. Transcanial Direct Current Stimulation 

tDCS was delivered using a constant-current stimulator (Soterix model 1224-B, Soterix Medical Inc., New York, NY, USA) via two (5 × 7cm) saline-soaked electrode pads. Care was taken to make electrode placements in the same location for all subjects for both sham and real stimulation. The anode was placed at the midline, oriented horizontally (7 cm side mediolaterally) to cover the leg M1 of both hemispheres, and centered on the midline in between the ‘hot spots’ for TA in each hemisphere. The hotspot varied between individuals and was usually ≤1cm posterior to the vertex. The reference cathode was also oriented horizontally and centered on the inion. Impedance was minimized through careful preparation of the scalp to give an optimal contact quality reading. Participants underwent either 20 min of anodal or 20 min of sham stimulation in a pseudo-random manner and were blinded to their treatment. During anodal stimulation, the intensity was set to 2 mA and current ramped up and down over a 10 s period at the beginning and the end of intervention. During sham stimulation, the current intensity ramped up to 2 mA for a period of 10 s before ramping down to zero for the remainder of the stimulation. This was repeated at the end of stimulation, to mimic the sensation of the current ramping down at the end of anodal tDCS. Investigators doing the TMS study were also blinded.

### 2.4. Electromyography

Electromyography (EMG) signals were recorded via disposable surface electrodes placed at the muscle belly of the TA and the lateral malleolus of each limb. The ground electrode was placed on the knee. EMG signals were amplified and filtered (bandwidth 5 Hz to 1 kHz) with a VIKING IVP (Nicolet, Viasys Healthcare, Warwick, UK). All signals were sampled at 2 kHz, visually displayed on-line, and stored for off-line analysis using a custom Java Analyser for Waveform Signals (JAWS) program. Peak to peak MEP amplitudes were measured from unrectified single traces.

### 2.5. Transcranial Magnetic Stimulation

A MAGSTIM 200 stimulator (Magstim, Whitland, UK) was used to deliver single-pulse TMS via a double-cone coil of 11 cm in diameter, orientated to induce current in the posterior-anterior direction in the cortex. The vertex was taken as the intersection between the interaural and nasion-inion lines and this was marked on the cap for reference. The hotspot was identified in each hemisphere, contralateral to each target TA muscle. The hotspot was taken as the scalp location where the peak-to-peak MEP amplitudes were greater in the target muscle than amplitudes of adjacent scalp locations for a given TMS stimulus intensity. The same hotspots were used throughout the assessment and rechecked between stimulus trials. Using a motor threshold assessment tool (an adaptive parameter estimation by sequential testing (PEST) procedure) [30,31] Resting Motor Threshold (RMT) was obtained for evoking an MEP of 0.05 mV peak to peak amplitude for the TA of each limb. A similar technique was used to obtain the stimulus required for evoking an MEP of 0.5 mV peak to peak for the TA of each limb (test stimulus). Test stimulus evoking MEP of 0.5 mV (rather than 1 mV) was chosen as it was difficult to get larger MEPs (1mV) in some subjects. MEPs were recorded 5, 10 and 15 min (T-5 to T-15) before the intervention (tDCS) and at 2, 5, 10, 20, 30 and 40 min after intervention (T2 to T40). Baseline MEP amplitude was taken as the average of the recordings (T-5 to T-15) before intervention. The mean MEP peak to peak amplitude for each TA was calculated from the average of 10 trials at each time point. Some trials were excluded due to increased background activity. Baseline averages were used to normalize post-intervention measures. Variability in TMS evoked MEPs was measured by calculating the coefficient of variation of baseline MEPs for each TA muscle (during each experiment). 

### 2.6. Statistical Analysis

Statistical analysis was performed using SPSS-software (SPSSv20 for Windows, SPSS Inc., Chicago, IL, USA). Shapiro-Wilk test was applied to check for normality of data. A three-way, repeated measures analysis of variance (ANOVA) was conducted with the factors: Side (left vs. right), stimulus (real and sham) and time (pre vs. post), with time as the repeated measure (baseline, T2–T40) on mean MEP amplitudes. Repeated measures ANOVA was also conducted separately on MEP amplitudes after real and sham tDCS. The Greenhouse–Geisser correction was used if necessary, to correct for non-sphericity. The effect of tDCS on each TA-MEP was assessed by a grand average of all normalized MEP values at time points T2–T40. Repeated measures ANOVA were performed for subgroups which showed increased or decreased grand average MEPs post tDCS. Post hoc paired *t*-tests (two-tailed) were used to evaluate changes in the size of the MEP response at different time points compared to baseline. Bonferroni corrections were made for multiple comparisons. Significance was set at *p* < 0.05. Independent sample *t*-tests were performed to compare baseline MEPs, variability of baseline MEPs (coefficient of variation), RMT, and test stimulus intensity between subgroups 1 and 2 (real or sham stimulation) 

## 3. Results

All subjects reported mild tingling or burning of the scalp at the beginning and end of stimulation. One subject reported headache after TMS. No other adverse effects were reported during or after tDCS. Subjects reported that they could not distinguish between the real and sham stimulation. Stimulation data and baseline evoked peak to peak MEPs are shown in Table 1. RMT ranged from 27–61% of maximum stimulator output (MSO) and Test Stimulus (TS) ranged from 30–78% of MSO. There were no significant differences in the RMT, TS or baseline MEP amplitude evoked between right and left hemisphere stimulation or between participants when undergoing real and sham stimulation (ANOVA).

### Comparison of Real and Sham tDCS Stimulation for 20 min

**Figure 1:**
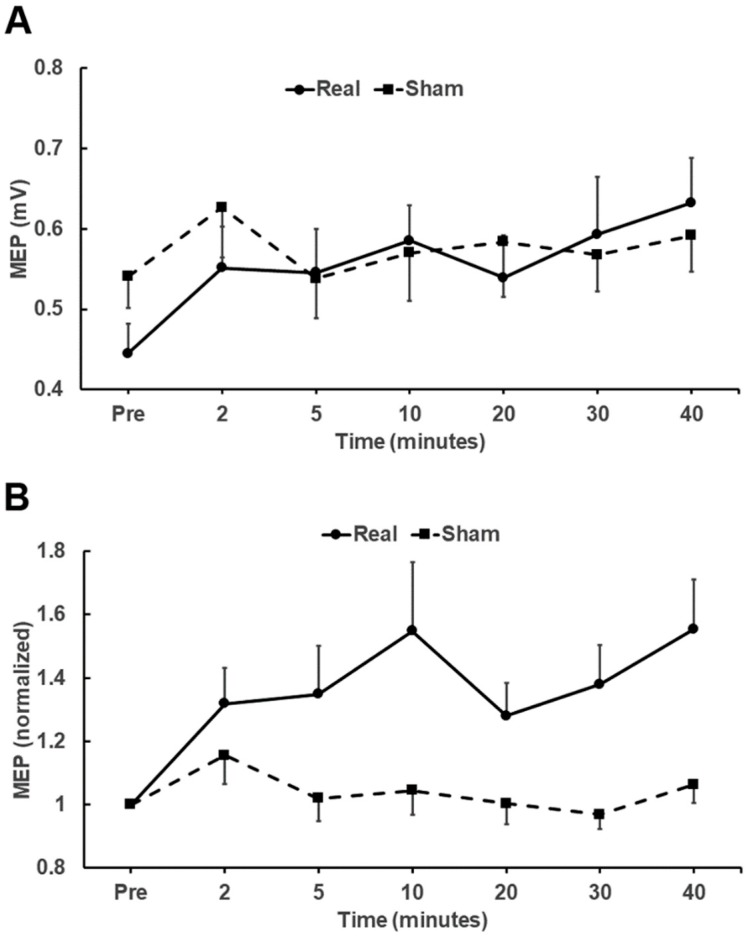
Raw (**A**) and normalized (**B**) mean MEP (± SEM) amplitudes (mV) recorded from the Tibialis Anterior muscle of both sides of all participants before and after 20 min of anodal stimulation (circles, uninterrupted line) and 20 min of sham stimulation (squares, dashed lines). MEP: Motor Evoked Potentials; SEM: Standard Error of Mean.

**Figure 2:**
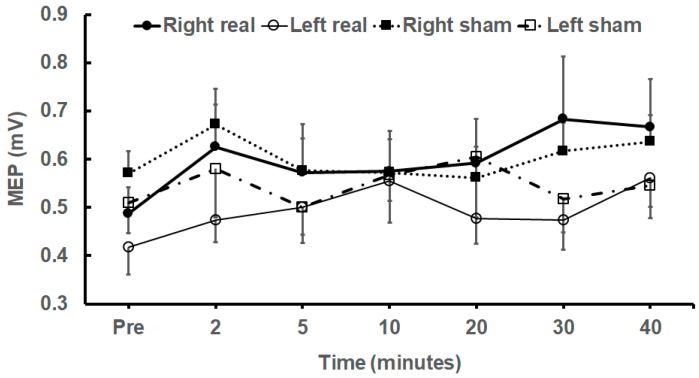
Mean MEP (± SEM) amplitudes (mV) recorded from the right (filled symbols) and left (open symbols) Tibialis Anterior muscles before and after 20 minu anodal (circles) and 20 min of sham (squares) tDCS.

Statistical analysis was performed for comparison of real and sham tDCS effects. The 3-way repeated measures ANOVA (comparing raw MEP amplitudes) using within-subject factors of stimulus (real/sham) and side (left/right), and time as the repeated measure revealed a significant effect of time (F_5.804, 258_, *p* = 0.023, η^2^*_p_* = 0.055) and interaction (time × stimulus) (*F*_5.331, 240_ = 2.192, *p* = 0.045, η^2^*_p_* = 0.052). There were no other significant interactions (time × side, time × stimulus × side). Figure 1A,B plot raw and normalized MEP (± Standard Error of Mean (SEM)) amplitudes, respectively, recorded from TA of both sides of all subjects following real and sham stimulation. Figure 2 plots the mean (± SEM) raw MEP data from the right and left TA after anodal and sham tDCS.

Analysis of raw MEP data after real tDCS using repeated measures ANOVA was significant (*F*_2.659, 85.414_, *p* = 0.009, η^2^*_p_* = 0.147) while similar analysis for sham tDCS was not (*F*_2.853, 84.658_, *p* = 0.293, η^2^*_p_* = 0.057). For the real tDCS data pairwise comparison revealed significant effects only at time points 30 and 40 min with corrections for multiple comparisons.

Grand averages of the normalized post tDCS response (grand averages of the mean MEPs at 2, 5, 10, 20, 30 and 40 min) for both TA muscles of all subjects after real tDCS are shown in Table 2. There is considerable variability in the extent and direction of modulation between subjects and hemispheres. As both excitatory and inhibitory effects have been seen from anodal tDCS of the leg motor area, the grand averages were used to subdivide the population into increased excitation (>1, group 1) and reduced excitation (<1, group 2) subgroups. 

**Figure 3:**
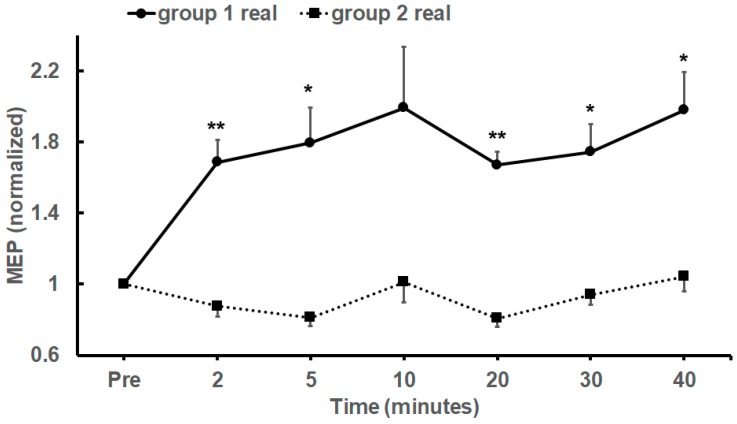
Normalized Mean MEP (± SEM) amplitudes recorded from of groups 1 (circles, uninterrupted line) and 2 (squares, dotted line) before and after 20 min of anodal tDCS. Post-hoc significance results are indicated by * (*p* < 0.05) or ** (*p* < 0.01).

Inspection of the group 1 and 2 subpopulations (Table 2, MEP values of group 1 is marked with ^†^) showed that 4 of the 11 participants who received real tDCS for 20 min showed increased mean MEP of both TAs after tDCS, 3 participants showed reduced mean MEP of both TAs, while the final 4 showed a mixed response (increased mean MEP in one TA and reduced MEP in the other). These normalized TA-MEP population mean responses are compared in Figure 3.

Statistical analysis of subgroups using repeated measures ANOVA of the normalized data showed a significant effect of time after anodal tDCS in the group 1 subpopulation (*F*_22.212, 24.332_, *p* = 0.028, η^2^*_p_* = 0.266) but there were no significant effects in the group 2 subpopulation (*F*_2.718, 21.305_, *p* = 0.176, η^2^*_p_* = 0.171). Post-hoc analysis showed significant increase in mean MEPs in the group 1 subpopulations at all time points except at time point 10 min after tDCS (Figure 3).

There was no significant difference (*t*-tests) between the baseline MEPs, variability of baseline MEPs, RMT, or test stimulus intensity between subgroups 1 and 2 (real or sham stimulation).

## 4. Discussion

The main finding of the study was that anodal tDCS of the midline vertex resulted in increased excitability of leg M1 of both hemispheres in only 4 of the 11 participants (36%). There was increased excitability of leg M1 in one hemisphere in 4 participants (36%), while there was no effect in either hemisphere is 3 participants (27%). This is the first study which has evaluated the changes in excitability in the leg M1 of both hemispheres following anodal tDCS over the midline vertex. Previous studies have reported similar inter-individual variability in the efficacy of different plasticity-inducing brain stimulation protocols including anodal tDCS, Paired Association Stimulation (PAS25) and Intermittent Theta Burst Stimulation (iTBS) [13,14,32]. Facilitation of MEPs was seen in 43–60% of participants (referred to as ‘responders’) after anodal tDCS of leg M1 of one hemisphere (by applying the stimulating electrode over the ‘hot-spot’ from which the largest MEPs were evoked in the contralateral TA) [15,23]. Similarly, facilitation of MEPs was only observed in 45–50% of participants after anodal tDCS of hand M1 [13,14]. 

Many studies have examined parameters that could predict efficacy of response following non-invasive brain stimulation protocols including tDCS. Most of these studies have evaluated excitability changes in the hand M1. Sensitivity to TMS (test stimulus intensity required to evoke 1 mV MEP or RMT) has been shown to correlate with tDCS efficacy by some studies [18,33] but not others [14]. We did not find any correlation between RMT, Test Stimulus Intensity, baseline MEP amplitude and baseline MEP variability with the efficacy of tDCS. This may be related to differences between leg M1 vs. hand M1 (with different anatomical orientation of neurons), and the fact that tDCS was applied to a location in between rather than over the hotspot for the targeted muscle. 

Other factors that are known to influence the induction of plasticity by non-invasive brain stimulation techniques include priming with stimulation, prior motor activity, and attention focus and behavioral engagement during stimulation [34]. It’s not clear if anodal tDCS is more effective when applied during a motor task. Anodal tDCS of the leg M1 was found to increase M1 excitability when applied during a skilled task [15], while anodal tDCS of the hand M1 was less effective when applied during a motor task [35] than at rest. A previous study showed greater efficacy of tDCS during gait training [5] and future studies should evaluate if this is related to greater excitability changes in leg M1s. Other factors that can be controlled when designing the studies include performing the trials at similar times of the day, ensuring that participants are alert during stimulation and excluding those taking central nervous system active drugs [34]. 

Differences in structure and electrical conductivity of the scalp, skull and meninges, and the orientation of sulci and neurons have been proposed to result in different susceptibility to applied currents, and variability of tDCS after effects [9,16]. Computational modeling is increasingly being used for rational design of electrode montages to calculate electric field strength and current flow direction in relation to neuronal orientation [16,36,37]. Foerster et al. [37] found the conventional electrode montage for anodal tDCS of leg M1 of one hemisphere (5 × 7 cm electrodes with anode over the hot spot and cathode over the contralateral supraorbital area) to be the most effective compared to other electrode montages. They also found that a smaller anode (3.5 × 1 cm) over the hot spot and cathode over T7 (10-10 EEG system) was equally effective. A similar analysis for targeting both leg M1s would be helpful in designing more efficient electrode montages. 

Effects of anodal tDCS in leg M1 have been evaluated using different electrode sizes and montages, as well as varying duration and intensities of stimulation. The active electrode (anode) has been applied lateral to the midline over the most effective site for evoking MEPs in the contralateral leg muscles [14,19,20] to facilitate the leg M1 of one hemisphere. Electrode sizes have varied between 8–35 cm^2^ in these studies. These studies have placed the cathode over the contralateral supraorbital region [14,21,22,23]. Other studies have applied anodal tDCS over the vertex (midline) and used larger electrode sizes (25–40 cm^2^) to facilitate leg M1 of both hemispheres [3,4,5,6,38]; the reference or cathodal electrode has been applied to the right supraorbital region [6], the middle of the forehead [38] or inion [3,4,5]. Stimulation intensities have varied from 1–2 mA and durations from 10–20 min. The effects of varying montages, durations and intensities have only been partly evaluated for the leg M1 [22,23,33,37]. Studies of tDCS applied to the hand motor area have shown greater excitability changes with larger electrode sizes [39]. Some studies have found greater efficacy with higher current intensity [22] while other studies found lower current intensities to be equally effective [33,39]. Cathodal stimulation over the inion could potentially affect cerebellar excitability. Kaski et al. [4] did not find any effect of this montage (anodal electrode over the vertex and cathodal electrode over the inion) on the conditioned eyeblink response. 

We used a 2 mA stimulus intensity (which results in a current density of 60 µA/cm^2^ when applied through a 35 cm^2^ electrode) for tDCS in our study as other studies have found consistent modulatory effects on the leg motor cortex with this intensity [4,22] and have shown it to be safe [40,41]. Only anodal stimulation was tested as it has been the preferred mode of stimulation for improving motor performance [3,4,5,6,42], and cathodal stimulation does not produce consistent modulatory effects on the leg area of the motor cortex [22,23,25]. The anode was placed over the vertex and cathode placed over the inion, as this configuration causes excitation of the leg motor cortex of both hemispheres [4] and has been shown to be effective in improving gait and balance [3,4,5].

Subdividing groups according to their grand average response removes information about the time course of tDCS effects. We examined the temporal pattern of all participants and there was no tendency of individuals to show any clear pattern of early or late excitation or inhibition. 

There are several limitations of the current study. The number of subjects was small and small differences in effects may not have been detected. We evaluated only 10 MEPs per time point before and after tDCS to assess corticospinal excitability and recent studies have advised larger numbers of MEPs (due to variability) [43]. This may have reduced our ability to detect excitability changes reliably. However, we believe that adequate sampling was performed as we examined MEPs 3 time points before and 5 time points after tDCS conditioning. There is some uncertainty about the blinding efficacy of sham tDCS stimulation [44]. As in most studies we delivered active stimulation for a short period at the beginning and at end of stimulation to mimic the skin sensations perceived during active stimulation. Participants were asked to guess if they had received real or sham stimulation and they said they were unsure.

There are many ways tDCS parameters can be combined, and more studies are needed to determine which stimulation paradigms are more effective in changing cortical excitability and improving motor or other neurological function. Transcranial direct current stimulation (with or without physical treatment) has been applied to the leg motor cortex to improve walking and balance following brain injury [2,3,41]. In chronic stroke patients, anodal tDCS was applied to the ipsilesional hemisphere to preferentially stimulate leg M1 of the affected side [2,41]. Anodal tDCS of the ipsilesional leg M1 was also found to inhibit the contralesional M1 in some participants [2] and this effect was also shown in healthy individuals [23]. Excitation of one hemisphere and simultaneous inhibition of the opposite hemisphere may be of benefit in stroke patients, where suppression of the contralesional M1 has been shown to improve paretic arm function [45,46,47]. However, this may not be the ideal stimulation paradigm for improving mobility and balance when both hemispheres may be affected (e.g., Parkinson’s disease, Cerebral leukoaraiosis). Our study shows that midline tDCS is better than applying tDCS to the ‘hotspot’ of one hemisphere if we aim to increase excitability of both leg M1s [23].

## 5. Conclusions

Our study confirms that there is considerable inter-individual variability in the effects of anodal tDCS on excitability of leg M1s. Many clinical trials of tDCS for improving balance and walking placed stimulation electrodes at the midline vertex or anterior to it to simultaneously facilitate leg M1 of both hemispheres [3,4,5,6]. While these studies have reported improvement of gait after tDCS, it’s not clear if there is variability in improvement between individuals and if it correlates with the excitability changes observed in the cortex of one or both hemispheres. This information may be useful to tailor tDCS (and other non-invasive brain stimulation interventions) to optimize improvement.

## Figures and Tables

**Table 1:** Baseline TMS data in all participants.

	Real Stimulation		Sham Stimulation	
	Left Hemisphere	Right Hemisphere	Left Hemisphere	Right Hemisphere
Number	11	11	11	11
RMT (%MSO, Mean ± SEM)	43 ± 3.1	42 ± 2.7	43 ± 2.2	42 ± 2.7
Test Stim (%MSO, Mean ± SEM)	52 ± 3.2	52 ± 3.3	52 ± 3.1	51 ± 4
Baseline TA MEP (mV, Mean ± SEM)	0.49 ± 0.05	0.42 ± 0.06	0.57 ± 0.05	0.51 ± 0.06

Abbreviations: MEP: Motor Evoked Potentials; MSO: Maximum Stimulator Output; RMT: Resting Motor Threshold; SEM: Standard Error of Mean; TA: Tibialis Anterior, TMS: Transcranial Magnetic Stimulation.

**Table 2:** Grand average of normalized mean MEP amplitude post real tDCS.

Participant	Real tDCS	
	Right TA	Left TA
1	0.99	0.94
2	1.80 ^†^	1.29 ^†^
3	1.89 ^†^	0.89
4	1.88 ^†^	2.13 ^†^
5	1.36 ^†^	1.50 ^†^
6	0.88	1.83 ^†^
7	0.97	0.86
8	0.88	0.87
9	0.96	1.31 ^†^
10	2.29 ^†^	1.72 ^†^
11	0.92	2.75 ^†^

Abbreviations: MEP: Motor Evoked Potentials; TA: Tibialis Anterior; tDCS: transcranial Direct Current Stimulation; ^†^ MEP values in subgroup 1.

## Abbreviations

M1	Motor cortex
MEP	Motor Evoked Potential
MSO	Maximum Stimulator Output
RMT	Resting Motor Threshold
TA	Tibialis Anterior
tDCS	Transcranial Direct Current Stimulation
TS	Test Stimulus
